# Profiling Cell Heterogeneity and Fructose Transporter Expression in the Rat Nephron by Integrating Single-Cell and Microdissected Tubule Segment Transcriptomes

**DOI:** 10.3390/ijms25053071

**Published:** 2024-03-06

**Authors:** Ronghao Zhang, Darshan Aatmaram Jadhav, Najeong Kim, Benjamin Kramer, Agustin Gonzalez-Vicente

**Affiliations:** 1 Department of Physiology and Biophysics, Case Western Reserve University School of Medicine, Cleveland, OH 44106, USA; 2 Department of Cell Biology, Emory University School of Medicine, Atlanta, GA 30322, USA; 3 Department of Biomedical Engineering, Georgia Institute of Technology & Emory University, Atlanta, GA 30332, USA; 4 Department of Kidney Medicine, Glickman Urological & Kidney Institute, Cleveland Clinic, Cleveland, OH 44106, USA

**Keywords:** hexoses, sugar transport, SGLT2 inhibitors, salt-sensitive hypertension

## Abstract

Single-cell RNA sequencing (scRNAseq) is a crucial tool in kidney research. These technologies cluster cells based on transcriptome similarity, irrespective of the anatomical location and order within the nephron. Thus, a transcriptome cluster may obscure the heterogeneity of the cell population within a nephron segment. Elevated dietary fructose leads to salt-sensitive hypertension, in part, through fructose reabsorption in the proximal tubule (PT). However, the organization of the four known fructose transporters in apical PTs (SGLT4, SGLT5, GLUT5, and NaGLT1) remains poorly understood. We hypothesized that cells within each subsegment of the proximal tubule exhibit complex, heterogeneous fructose transporter expression patterns. To test this hypothesis, we analyzed rat kidney transcriptomes and proteomes from publicly available scRNAseq and tubule microdissection databases. We found that microdissected PT-S1 segments consist of 81% ± 12% cells with scRNAseq-derived transcriptional characteristics of S1, whereas PT-S2 express a mixture of 18% ± 9% S1, 58% ± 8% S2, and 19% ± 5% S3 transcripts, and PT-S3 consists of 75% ± 9% S3 transcripts. The expression of all four fructose transporters was detectable in all three PT segments, but key fructose transporters SGLT5 and GLUT5 progressively increased from S1 to S3, and both were significantly upregulated in S3 vs. S1/S2 (*Slc5a10*: 1.9 log2FC, *p* < 1 × 10^−299^; *Scl2a5*: 1.4 log2FC, *p* < 4 × 10^−105^). A similar distribution was found in human kidneys. These data suggest that S3 is the primary site of fructose reabsorption in both humans and rats. Finally, because of the multiple scRNAseq transcriptional phenotypes found in each segment, our findings also imply that anatomical labels applied to scRNAseq clusters may be misleading.

## 1. Introduction

Single-cell RNA sequencing (scRNAseq) technologies cluster cells based on transcriptome similarity, without considering the anatomical arrangement critical for kidney tubular epithelial cell functions. Such clusters are associated with distinct segments of the nephron, based on the expression of marker genes characteristic of those segments. Previous studies have suggested the presence of functionally distinct, intermingled cell types in specific nephron segments [1]; thus, a transcriptional cluster comprised only of cells with highly similar transcriptional phenotypes may not accurately represent the entire cell population within a specific region of the nephron. To explore the association between single-cell cluster transcriptional phenotypes and anatomical localization, we compared transcriptomes from scRNAseq (dispersed cells) with those from manually microdissected rat nephron segments (anatomical context).

Modern Western diets are rich in sodium chloride (NaCl) and fructose, or fructose-containing syrups [2], while in Asian countries such as China consumption of sugar-sweetened beverages has doubled within the last decade [3,4]. As such, the average calories ingested from fructose in industrialized countries expressed as a percentage of the recommended average caloric intake exceeds 10% [5,6,7]. Importantly, individuals consuming more than 74 g/day of fructose, equivalent to ~15% of calories on a 2000 kcal/day diet, have higher blood pressure [8]. Previous studies have shown that rodents consuming 10 to 20% of their calories as free-fructose develop salt-sensitive hypertension within a week [9,10,11,12,13]. In addition, in chronic models of metabolic syndrome, such as those where experimental diets contain nearly 50% of dietary fructose by weight, restricting Na^+^ intake ameliorates the increase in blood pressure [13,14]. These findings provide a connection between the ingestion of fructose and salt and the development of hypertension.

Fructose undergoes glomerular filtration, and the proximal tubule (PT) reabsorbs the bulk of it. Four apical transporters with vastly different kinetic properties could transport fructose: SGLT5 (fructose K_m_: 0.62 mmol/L), NAGLT1 (K_m_: 7.8 mmol/L), SGLT4 (K_m_ > 10 mmol/L), and GLUT5 (K_m_: 12.6 mmol/L). Some of these transporters also exhibit the ability to transport glucose, for which the two monosaccharides may compete (Supplemental Table S1). Furthermore, the distribution of fructose transporters varies along the three subsegments of the PT [15], for which the contribution of each subsegment to overall fructose transport remains poorly understood. The PT is also the only nephron segment with high expression of fructokinase, an intracellular enzyme that phosphorylates fructose to fructose-1-P, thereby sequestering it within the cell [16,17]. Thus, unlike glucose which is hardly metabolized by PTs and is returned into the blood, fructose is broken down into 3-carbon intermediates which are metabolized by glycolysis or gluconeogenesis, or synthesized to diacylglycerol and neutral lipids [18]. Importantly, fructokinase is not regulated by substrate or product availability [16,17], for which an increase in intracellular fructose could therefore deplete ATP, and lead to inflammation and endothelial injury [19,20].

Our study challenges the dogma that fructose is uniformly reabsorbed along the pars-recta of the proximal tubule. Rather, we hypothesize that cells within each subsegment of the proximal tubule exhibit complex, heterogeneous fructose transporter expression patterns, which impact the fructose transport capacity. To explore this hypothesis, we extensively analyzed publicly available resources and integrated kidney single-cell transcriptomes with bulk transcriptomic data from micro-dissected kidney regions.

## 2. Results

### 2.1. Single-Cell RNAseq Map of the Rat Kidney

**Figure 1:**
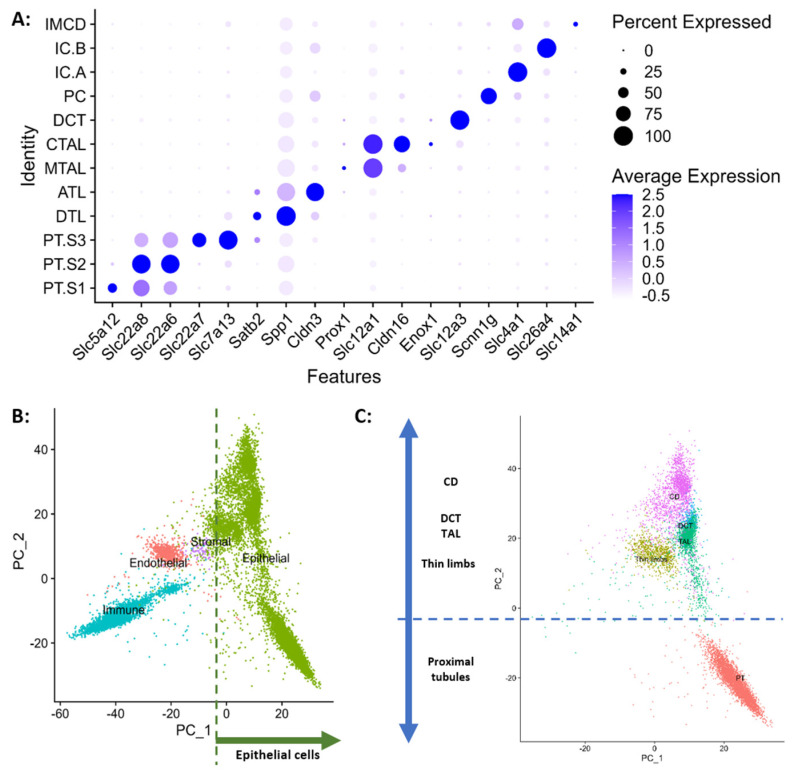
Cell type assignments and principal component analysis (PCA) projections of rat kidney cell clusters. (**A**) Cell type annotation using HuBMAP Kidney v1.2 cell markers. (**B**) PCA projections show that Epithelial cells (green) separate from other cell classes (Immune: cyan, Endothelial: pink and Stromal (purple) along PC1, and (**C**) PCA projections show tubular epithelial cells separate along PC2 resembling the anatomy of the nephron (Proximal Tubules: pink, Thin Limbs: army green, Thick Ascending Limbs: green, Distal Convoluted Tubule: cyan and Collecting ducts: purple).

Using publicly available scRNAseq transcriptomes from rat whole kidneys and a curated collection of tubular epithelial cell markers [21,22,23], we successfully identified different cell clusters. These encompass proximal tubules (PT) with segments S1, S2, and S3, descending thin limbs (DTL), ascending thin limbs (ATL), thick ascending limbs in both medullary (MTAL) and cortical (CTAL) regions, distal convoluted tubules (DCT), and cells from the connecting tubule and collecting duct system. These cells include principal cells (PC), intercalated cell types A and B (IC.A and IC.B), and inner medullary collecting duct cells (IMCD) (Figure 1A). Principal components analysis (PCA) projections of all cell classes show that the tubular epithelium separates from other cells along the first principal component (PC1) (Figure 1B), while PT cells separate from all other epithelial cells along the second principal component (PC2) (Figure 1C). Moreover, the distribution of tubular epithelial cells along PC2 resembles the anatomy of the nephron with the more distal cells exhibiting the greater separation from PT cells. As a final quality check, we contrasted our rat kidney map annotations with bulk transcriptomes from manually micro-dissected rat tubules (Appendix A in Figure A4) and with the subregional transcriptomes obtained from the KPMP (Appendix A in Figure A5), demonstrating great similarity between published rat and human transcriptomes annotations.

### 2.2. Cellular Heterogeneity in Different Nephron Segments

We next estimated the proportion of the distinct cell transcriptional phenotypes identified by scRNAseq within each segment of the nephron. We found that PT.S1 segments primarily consist of S1 cells (81% ± 12%) with a small proportion of S2 cells (12% ± 11%). Micro-dissected PT.S2 segments contain a mix of S1 (18% ± 9%), S2 (58% ± 8%), and S3 (19% ± 5%) cells. PT.S3 segments were primarily composed of S3 cells (75% ± 9%), with lower proportions of the S2 (10% ± 5%) and S1 (8% ± 7%) cell types. The thin portions of the Loop of Henle displayed a high percentage of the corresponding cell type: outer medullary DTL (OMDTL) (91% ± 2%, DTL), inner medullary DTL (IMDTL) (86% ± 3% DTL), and ATL (78% ± 9% ATL). Micro-dissected MTAL segments were a mix of MTAL (25% ± 15%) and CTAL (69% ± 15%) cells, while micro-dissected CTAL segments were almost exclusively composed of CTAL cells (95% ± 2%). DCT segments exhibited a majority (80% ± 5%) of DCT cells with some principal (2% ± 1%) and intercalated (1% ± 1%) cells. In CNT, CCD, and OMCD, we observed a continuous increase in the abundance of PC cells (58% ± 6%, 75% ± 3%, and 78% ± 6%, respectively) along with a decrease in IC (32% ± 2%, 20% ± 3%, and 15% ± 4%, respectively). Micro-dissected IMCD segments contained PC (51% ± 7%), IMCD (27% ± 11%), IC (4% ± 1%), and a significant percentage of ATL (17% ± 4%) cells, which may reflect contamination (Supplemental Table S2, Summary).

### 2.3. Transport and Metabolism of Fructose

**Figure 2:**
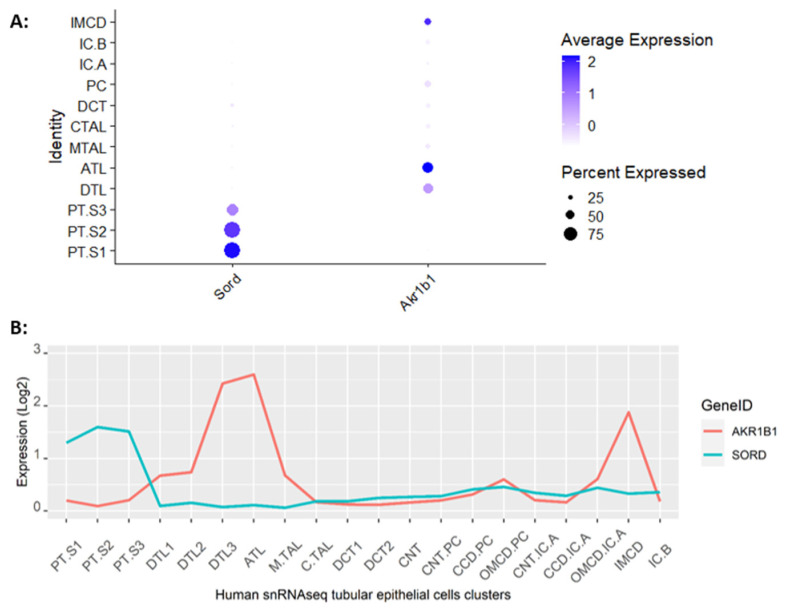
Expression of enzymes from the sorbitol pathway. (**A**) A dot plot of rat scRNAseq data show that sorbitol dehydrogenase (Sord), which converts sorbitol to fructose, is predominantly expressed in proximal tubules, while aldolase reductase (Akr1b1), which converts glucose to sorbitol, is predominantly expressed in medullary segments. (**B**) Analysis of human snRNAseq transcriptomes shows that the expression of aldolase reductase (AKR1B1) and sorbitol dehydrogenase (SORD) genes in the human kidney resembles that of the rat.

We next studied the expression of sugar transporters and fructose metabolic enzymes. Fructose can be produced endogenously from glucose via the polyol pathway. We found that the expression of sorbitol dehydrogenase (Sord), the initial enzyme in the polyol pathway was stronger in PTs, while mRNA encoding aldose reductase (Akr1b1), the rate-limiting step in the polyol pathway, was predominantly expressed in medullary segments (Figure 2A). The distributions of these enzymes in rat match that in the human kidney (Figure 2B) [22,24].

**Figure 3:**
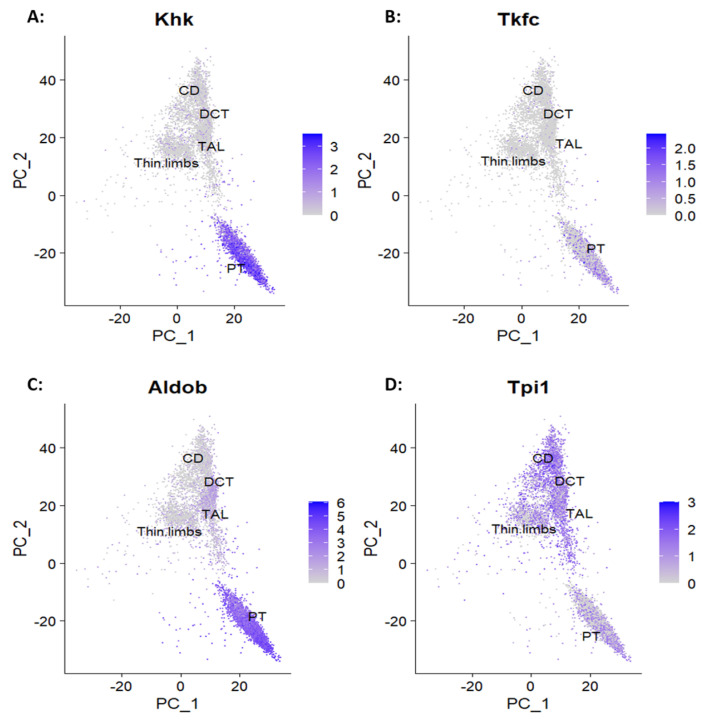
Expression of enzymes necessary to metabolize fructose into synthetic pathways. (**A**,**B**) Enzymes specific to fructose metabolism fructokinase (Khk) and triokinase (Tkfc) are mostly restricted to proximal tubules. (**C**,**D**) The enzymes shared with glycolysis and gluconeogenesis, aldolase B (Aldob), and triosephosphate isomerase (Tpi1) are widely expressed in other segments in addition to proximal tubules.

Then, we examined the expression of enzymes allowing fructose metabolites to enter glycolytic pathways and neutral lipids synthesis [25,26]. Fructokinase (Khk) and triokinase (Tkfc) are specific to fructose metabolism and are restricted to PTs, indicating that this segment metabolizes the bulk of fructose (Figure 3A,B). On the contrary, aldolase B (Aldob) and triosephosphate isomerase (Tpi1), which also participate in glycolysis and gluconeogenesis, present a widespread anatomical distribution (Figure 3C,D).

Next, we extracted the PT clusters and conducted differential gene expression analysis between PT.S1, PT.S2, and PT.S3 to identify significant differences in the expression of fructose transporters and metabolic enzymes (Supplemental Table S3).

**Figure 4:**
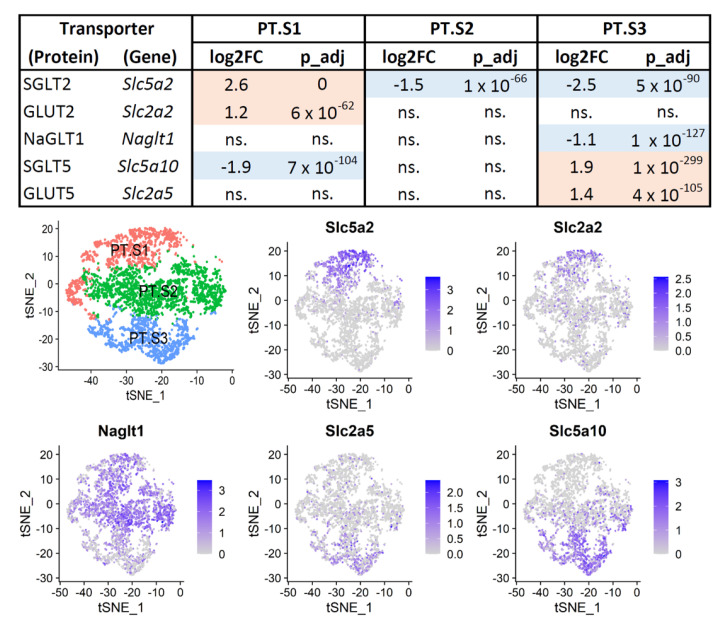
Sugar transporters are differentially expressed across proximal tubule cell types. (**Upper table**) log2 fold change (Log2FC) and *p* values on each segment, as compared to the other two segments together. (**Lower panels**) t-distributed stochastic neighbor embedding (tSNE) projections of proximal tubule clusters showing a transcript density map of the differentially expressed sugar transporter in rat proximal tubule scRNAseq clusters. Only differentially expressed sugar transporters are shown in the figure. The tSNE analysis was run in all cells (Appendix A in Figure A1D) and then the clusters corresponding to proximal tubules were extracted for visualization. Proximal tubule cells were not reclustered to create this figure.

Several genes were differentially expressed in each PT cluster as compared to the other two together, including fructose and glucose transporters (Figure 4). Both SGLT2 (*Slc5a2*) and GLUT2 (*Slc2a2*) were upregulated in PT.S1 while SGLT5 (*Slc5a10*) was downregulated. The PT.S2 cluster only presented downregulation of the SGLT2 gene (*Slc5a2*). Finally, SGLT5 (*Slc5a10*) and GLUT5 (*Slc2a5*) were both upregulated in PT.S3, while SGLT2 (*Slc5a2*) and NAGLT1 (Naglt1) were downregulated in this cluster. These data indicate that the pairs SGLT5/GLUT5 and SGLT2/GLUT2 are transcriptionally paired in rat PT cells. SGLT1 (*Slc5a1*), GLUT1 (*Slc2a1*), and SGLT4 (*Slc5a9*) were not differentially expressed in any of the three PT clusters; however, there were smaller differences in expression below the significance threshold (Appendix A in Figure A6) across clusters. Gene transcripts of enzymes participating in the polyol pathways were not differentially expressed across clusters.

**Figure 5:**
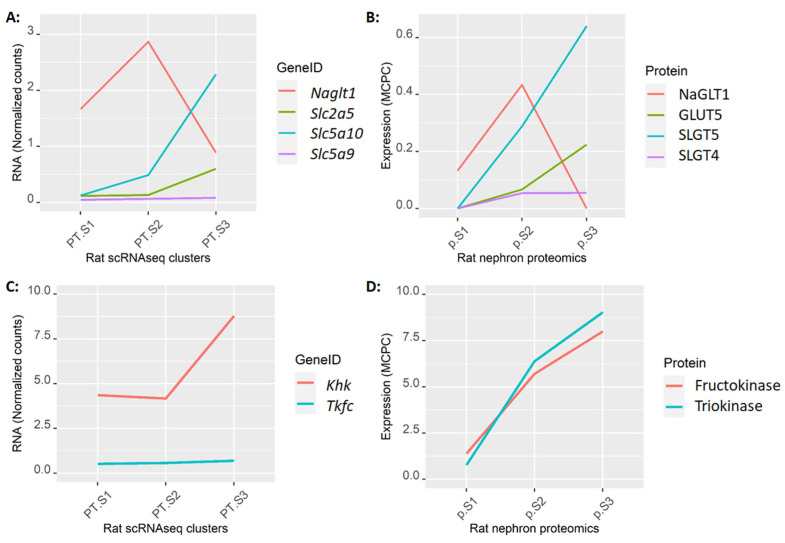
Normalized RNA transcripts of fructose transporters in rat scRNAseq proximal tubule clusters (**A**) compared to their corresponding proteins expressed in “million copies per cell” (MCPC) in microdissected rat proximal tubule segments (**B**). Normalized RNA transcripts of fructokinase (Khk) and Triokinase (Tkfc) in rat scRNAseq proximal tubule clusters (**C**) compared to their corresponding proteins expression in microdissected rat proximal tubule segments (**D**). The order and colors of GeneIDs on the left panels (**A**,**C**) match that of the corresponding protein on the right panels (**B**,**D**).

Finally, to qualitatively assess whether RNA transcript abundance resembles differences in protein expression, we examined the protein expression of fructose transporters and metabolic enzymes in micro-dissected rat PT segments and compared them with the RNA expression in rat scRNAseq (Figure 5). Importantly, all four fructose transporters were expressed in both S2 and S3, and fructose transporter mRNA and protein expression tightly paralleled one another. A similar analysis of glucose transporters (Appendix A in Figure A7) as well as the transcript levels in human snRNAseq data (Appendix A in Figure A8) can be found in the Appendix A. Although subtle differences were observed between rat and human fructose transporter expression, fructokinase, and triokinase expression were remarkably similar between rat and human proximal tubule segments.

## 3. Discussion

To study the distribution of fructose transporters and metabolic enzymes along the nephron we constructed a scRNAseq map of rat kidneys, using cell annotations aligned with those in humans. The projection of principal components highlights the separation of the tubular epithelium from other cell classes along PC1, while the positioning of epithelial cells along PC2 corresponds to the anatomy of the nephron, with proximal and distal cells exhibiting greater separation.

An important limitation of single-cell sequencing is the lack of spatial information. For instance, in scRNAseq analysis, cells are grouped based on transcriptional similarities, disregarding anatomical or spatial context. Thus, we established connections between cell clusters, as defined by the expression of marker genes, and nephron anatomy in two different ways. Firstly, we assessed the similarity between the average gene expression in scRNAseq clusters and the bulk gene expression in micro-dissected tubule segments. Secondly, we deconvoluted bulk transcriptomes from micro-dissected nephron segments using scRNAseq cellular cluster transcriptomes as a reference. Both approaches yielded consistent evidence for the coexistence of multiple cell types in different subsegments of the nephron. In PTs, both S1 and S3 present more than 75% of their corresponding cell type; however, S2 presents 58% of S2 cells with the remaining cells from adjacent S1 and S3. This suggests a differentiation continuum from S1 to S3 with diffuse anatomical boundaries around S2. In contrast to PTs, the thin portion of the loop of Henle (DTL and ATL) exhibits high correlation and cell percentages of the respective cell clusters, suggesting high homogeneity of transcriptional phenotypes with well-defined anatomical transitions. We identified two different cell types that map to the thick portion of the loop of Henle, the MTAL phenotype restricted to the MTAL, and the CTAL phenotype present in higher abundance in both subsegments. These cell types may correspond to the medullary “smooth surface” and cortical “rough surface” cells described in rat TAL [27,28,29]. Microdissected DCT presented a majority of DCT cells expressing NCC (*Slc12a3*) [1]; however, bulk DCT transcriptomes correlated with the DCT and TAL clusters. The reason could be twofold. On one hand, genes highly expressed in TAL like uromodulin (Umod) or those involved in Mg^++^ reabsorption are also expressed in the initial DCT [1,30], on the other hand, the rat TAL extends beyond the macula densa [31] which could confound dissection.

Finally, our data indicate that PC is the most abundant cell type in distal tubules, increasing their abundance along CNT, CCD, and OMCD, while IC presents an opposite pattern. The PC phenotype was also the most abundant cell type on IMCD, followed by 27% of IMCD cells. These cells express urea transporters and exhibit some transcriptional characteristics of both, principal and intercalated cells. In summary, our analysis is consistent with the well-established cell heterogeneity in connecting tubules and the collecting duct system.

The insights generated from this analysis present limitations inherent to the type and quality of the data. An important limitation is the lower number of cells and sequencing depth as compared to newer scRNAseq rat datasets [32,33]. We are also aware that experiments counting a higher number of cells had identified not only more kidney cell types but also different differentiation stages [22]. In addition, even though our efforts to identify contaminations with adjacent tubules, it is possible that the micro-dissected segments contain some cells from other regions as it is difficult to define morphological boundaries. Still, with these limitations, we can indicate that the terminology used in scRNAseq cluster assignment referring to specific nephron segments is misleading, as it encompasses anatomical regions with multiple coexisting cell types. Other investigators arrived at similar conclusions by deconvoluting rat kidney spatial transcriptomes [32]. Together, these data highlight the importance of interpreting single-cell data within the cellular spatial context in applications such as the mathematical modelling of nephron transport processes or the study of cell–cell interactions.

The consumption of moderately enriched fructose diets has been associated with the development of salt-sensitive hypertension, in part, due to its actions in the kidney [34,35,36,37,38]. Thus, once we established the scRNAseq map of the rat kidney, we focused on the study of genes involved in fructose transport and metabolism. We first looked at the polyol pathway, which is the only metabolic pathway known to produce fructose in humans [39]. Transcript levels of aldolase reductase in humans and rats indicate that sorbitol production occurs primarily in the kidney medulla, consistent with the necessity to produce an intracellular osmolyte for protecting cells in a hypertonic environment [40,41]. On the contrary, the sorbitol dehydrogenase transcripts found in PTs indicate that sorbitol will be converted to fructose in this segment, thereby feeding endogenous fructose into metabolic pathways [42,43].

The pars recta of the PT reabsorbs the bulk of fructose from the luminal fluid. We previously showed that in isolated-perfused rat S2 segments, Na^+^ removal from the luminal perfusate blunts fructose reabsorption by 86% [44]. This indicates that fructose reabsorption in PT.S2 is Na^+^-dependent, but the information in the S3 and S1 segments was missing. Here, we use transcript levels and proteomics data to predict the contribution of each subsegment of the proximal tubule to the overall reabsorption of fructose. Consistent with previous studies reporting congruent transcripts and protein levels along the rat nephron [45], we found that the protein expression of both fructose transporters in microdissected segments was consistent with RNA expression in scRNAseq PT clusters.

Four apical transporters could transport fructose in PT: NAGLT1, SGLT4, GLUT5, and SGLT5. Transcripts and protein levels of SGLT5 and GLUT5 were higher in the S3 portion of the rat PT as compared to S1 and S2. SGLT4 presented low gene and protein expression throughout PT subsegments, while NAGLT1 was primarily expressed in S2 and S1. This expression pattern reflects that of human orthologs.

In addition to transporter expression, an approximation of luminal fructose concentration and kinetic data are needed to estimate fructose transport. Recent calculations have estimated that, on average, healthy human kidneys filter 4 to 25 g of fructose per day, an amount corresponding to ~8% of the filtered glucose [46]. The percentage of ingested fructose that reaches the circulation after the first splanchnic extraction ranges from 15 to 50% [47]. Circulating plasma fructose is primarily metabolized by the liver and kidneys [48,49,50,51] with the latter accounting for up to 20% of fructose clearance from the plasma [51]. Unlike fasting plasma glucose, which is strictly maintained in the mmol/L range by several hormones, fasting fructose concentrations in humans are kept below 20 µmol/L [47,52]. Ingestion of a fructose-containing meal can raise plasma fructose concentrations more than 15 times, reaching values between ~30 to 300 µmol/L in humans [47,52,53,54] and ~50 to 200 µmol/L in mice [49,55]. Thus, even considering that proximal tubules reabsorb 70% of the luminal fluid, the intraluminal fructose concentrations are not expected to rise above 1 mmol/L.

NAGLT1, SGLT4, and GLUT5, all have affinities above 1 mmol/L [50,54,56,57,58,59,60], which dispute the contribution of these three transporters to the overall fructose reabsorption by the PT epithelia. GLUT5, in particular, has a reported Km of 6–9 mmol/L [54,58,59], and unlike the other two does not couple Na^+^ to energize transport [54,58,59]. On the contrary, SGLT5 has an affinity for fructose in the mid-µmol/L range and transports sugars with a 1:1 coupling ratio with Na^+^ [60,61,62]. Together with the expression data, this indicates that SGLT5 is the largest contributor to the overall reabsorption of fructose. In fact, SGLT5 localizes to the luminal membrane of the rat PT [44], and *Slc5a10* (-/-) mice given a fructose-rich diet excrete fructose in the urine [49].

Other investigators have reported that fructose stimulates the solute carrier family 12 member 1 (*Slc12a1*, NKCC2) in thick ascending limbs [63], and the solute carrier family 12 member 3 (*Slc12a3*, NCC) in the distal convoluted tubule [64]. Given the low luminal fructose concentrations in the ultra-filtrate and the fructose absorptive capacity of proximal tubules, it is unclear how much fructose could reach the distal nephron. In the current study, we were unable to detect the expression of fructokinase or fructose transporters above background levels outside the PT. Yet, it is possible that the polyol pathway acts as a source of interstitial fructose that can either diffuse through the paracellular pathway or enter cells basolaterally via GLUT2 in distal cells.

The scRNAseq data presented here were obtained using a balanced experimental design with pools of animals of different ages subjected to either ad libitum or restricted diet regimes [65]. However, this design does not include metabolic conditions that could affect fructose metabolism. For instance, chronic fructose feeding increases the expression of fructose transporters and fructose transport rates in PTs [44]. In addition, hyperglycemic and hypertonic conditions in diabetes are known to stimulate aldolase reductase [66], which given the expression of sorbitol dehydrogenase in PT would increase the flux of fructose into metabolic routes [20,42,43,67]. Given the significance of fructose metabolism in the development of metabolic syndrome and salt-sensitive hypertension, future studies should address these points.

## 4. Materials and Methods

### 4.1. Data Analysis

Unless otherwise noted, all data were analyzed in R: A language and environment for statistical computing and graphics (https://www.R-project.org/). Some data inspection, cleaning, or formatting were conducted in Notepad++ (https://notepad-plus-plus.org/) or Microsoft Excel 2016.

### 4.2. Rat Kidney Single-Cell Transcriptomes

Rat whole kidney single-cell RNA sequencing transcriptomes (scRNAseq) were obtained from the Gene Expression Omnibus (GSE137869). We used filtered_features_bc_matrix files from three pools of male (GSM4331828, GSM4331829, GSM4331830) and three pools of female (GSM4331831, GSM4331832, GSM4331833) Sprague Dawley rats. To generate these matrices, sequences from the microfluidic droplet platform were de-multiplexed and aligned to the rat genome (Rnor_6.0) using CellRanger (2.2.1) with default parameters [65]. Standard protocols were implemented in Seurat (4.3.0) for quality control and data analysis. In brief, a Seurat object was created from each filtered_features_bc_matrix after selecting features detected in at least three cells and cells containing at least 200 features. All six objects were merged yielding 19,414 cells with 16,821 features. Then the following filters were applied: nFeature_RNA > 560, nFeature_RNA < 4500, nCount_RNA < 30,000, percent.MT < 40, percent.Ribosomal < 30, and percent.Largest.Gene < 25. Filtered data were log-normalized. Cell-cycle scores were calculated using the cc.genes.updated.2019 genes and regressed during data scaling [68,69]. DoubletFinder (2.0.3) was used to remove doublets with parameters optimized with the function paramSweep_v3(). The entire quality control process removed ~30% of observations, yielding 16,821 features across 13,596 cells. After quality control, individual runs were split and SCTransform was applied. Then 3000 integration features were identified and all datasets were integrated using the IntegrateData() function. PCA identified 30 relevant dimensions for downstream processing. The function FindClusters() identified 36 clusters at a resolution of 1.6. The function FindMarkers() with the Wilcox test was used to identify cluster markers. Pseudobulk transcriptomes were obtained using the function AverageExpression().

### 4.3. Human Kidney Single-Nucleus Transcriptomes

An h5Seurat file containing single-nucleus RNA sequencing (snRNAseq) data was downloaded from the Kidney Precision Medicine Project (KPMP) tissue atlas (atlas.kpmp.org (accessed on 13 April 2023)) using the following filters: Experimental Strategy: Single-cell RNA-Seq, Workflow Type: Aggregated Clustered Data, File Name: c798e11b-bbde-45dd-bd91-487f27c93f8f_WashU-UCSD_HuBMAP_KPMP-Biopsy_10X-R_12032021.h5Seurat. The file contains aggregated cluster data including 30,395 features across 110,346 cells as described by Lake et al. [22]. In the KPMP Seurat object the aggregated clusters are annotated by: (1) “class” (cell Class), i.e., Epithelial, Stromal, Immune, Endothelial, or Neural, (2) “subclass.l1” (subRegion), i.e., podocytes (POD), parietal epithelial cells (PEC), PT, descending thin limb (DTL), ascending thin limb (ATL), thick ascending limb (TAL), distal convoluted tubule (DCT) connecting tubule (CNT), principal cell (PC) and intercalated cell (IC), Immune (IMN), fibroblasts (FIB), neural (NEU), papillary (Pape) and vascular smooth muscle/pericyte (VSM/P), and Endothelial (EC) cells, and (3) “subclass.l2” (cell type). Pseudobulk transcriptomes were obtained using the function AverageExpression() for cell Class and subRegion.

### 4.4. Rat Microdissected Tubule Segments Transcriptomes

To map single-cell transcriptional phenotypes with spatial and structural features of the nephron, publicly available bulk transcriptomes (PRJNA24440) from rat microdissected nephron segments [70] were downloaded from the National Center for Biotechnology Information (NCBI) Sequence Read Archive using SRA Toolkit 3.0.2. All raw sequencing FASTQ files were reprocessed using command lines in Ubuntu 22.04. In brief, quality control was performed on all files using FastQC v0.11.9 [71], and low-quality reads were trimmed using Trimmomatic v0.39 [72]. The remaining sequences were aligned to the rat genome obtained from the NCBI RefSeq Database (GCF_015227675.2_mRatBN7.2_genomic.fna.gz) using Burrows–Wheeler Aligner (BWA) v0.7.17-r1188 [73]. The alignment was then sorted and exported by Samtools v1.13 [74] in the format of BAM files. Reads were counted from BAM files using the R package Rsubread v2.12.0 with reference to genome annotation obtained from RefSeq (GCF_015227675.2_mRatBN7.2_genomic.gtf). The raw-counts matrices and metadata indicating the anatomical location of microdissected segments were processed and incorporated into a DESeq2 object (DESeq2 v1.34.0) [75]. Sequencing depth was normalized using the median of ratio method.

### 4.5. Correlation Analysis

Pseudobulk transcriptomes from individual rat scRNAseq clusters, regions, and cell types were contrasted with different transcriptomes from rats and humans, as well as with rat tubular proteomics data. For these comparisons we used genomewide Pearson correlation, as implemented by the function cor(x, method = “Pearson”, use = “pairwise.complete.obs”) in R. For visualizations, we used the function pheatmap(x) from the R package pheatmap_1.0.12.

### 4.6. Transcriptional Clusters Identity Assignment

**Figure A1:**
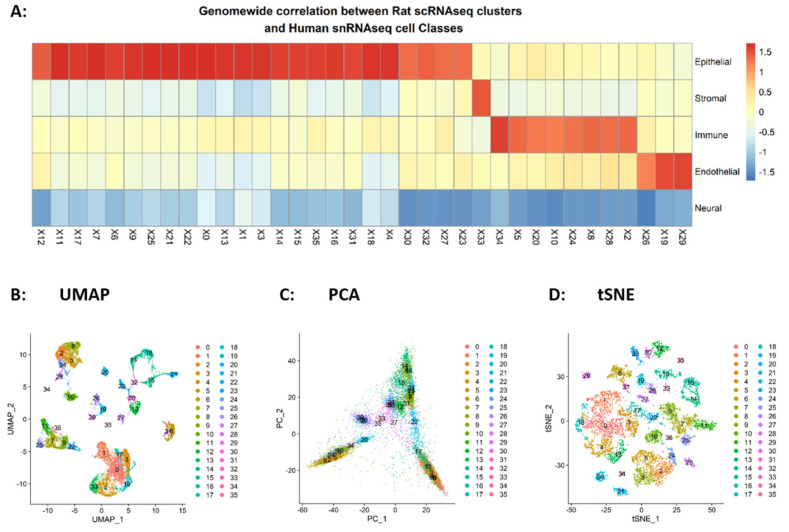
All rat scRNAseq clusters identified in the rat kidney were assigned to different cell classes by conducting a genomewide Pearson correlation with pseudobulk transcriptomes from human snRNAseq cell classes. (**A**) Normalized Pearson correlation coefficient between rat scRNAseq clusters (horizontal axis) and human snRNAseq cell classes (vertical axis) yielded: (1) Epithelial, 24 clusters; (2) Stromal, 1 cluster; (3) Immune, 8 clusters; and (4) Endothelial, 3 clusters, while no cluster in the rat dataset correlated with neural cells. (**B**) Uniform manifold approximation and projection (UMAP) of individual cell clusters. (**C**) Principal component analysis (PCA) projection. (**D**) t-distributed stochastic neighbor embedding (tSNE) projection of individual clusters.

**Figure A2:**
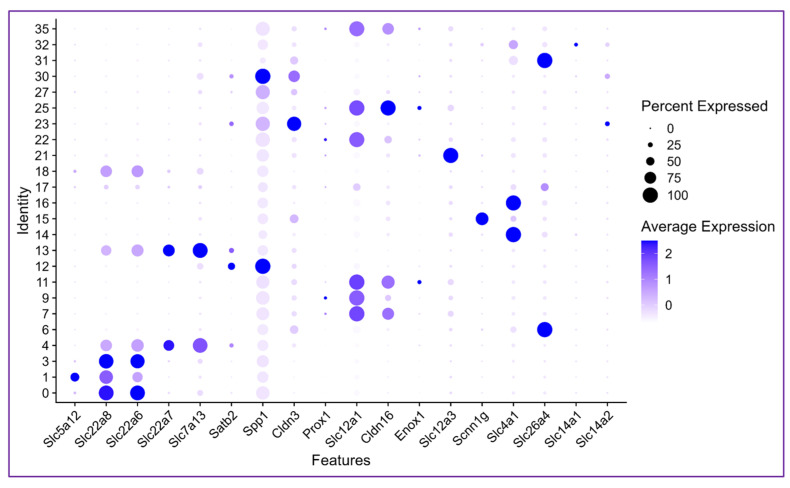
The tubular epithelial cell cluster identities were annotated using a curated list of cell markers from HuBMAP Kidney v1.2. See, the main manuscript for details.

**Figure A3:**
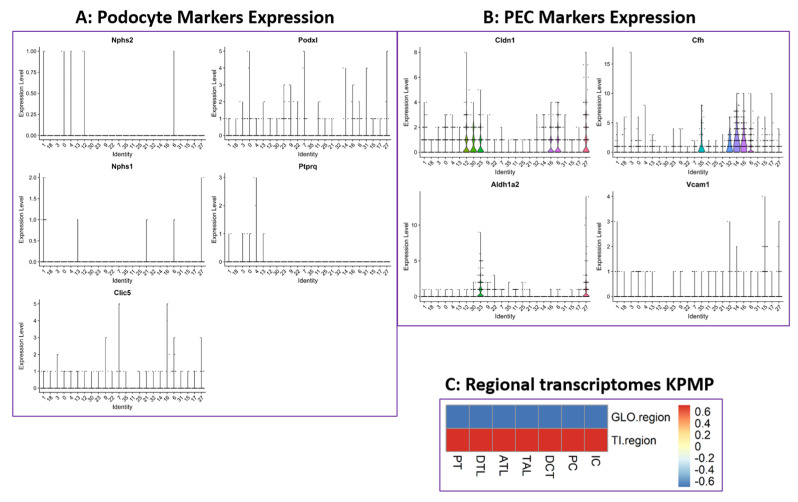
Cluster expression of gene markers for glomerular visceral epithelial cells. (**A**) Podocytes and (**B**) glomerular parietal epithelial cells (PEC). (**C**) Normalized Pearson correlation between rat scRNAseq cell clusters and human regional transcriptomes from the glomerular and tubulointerstitial regions (GLO.region and TI.region, respectively). Publicly available human kidney regional transcriptomics data were obtained from the Kidney Tissue Atlas (atlas.kpmp.org (accessed on 13 April 2023)).

**Figure A4:**
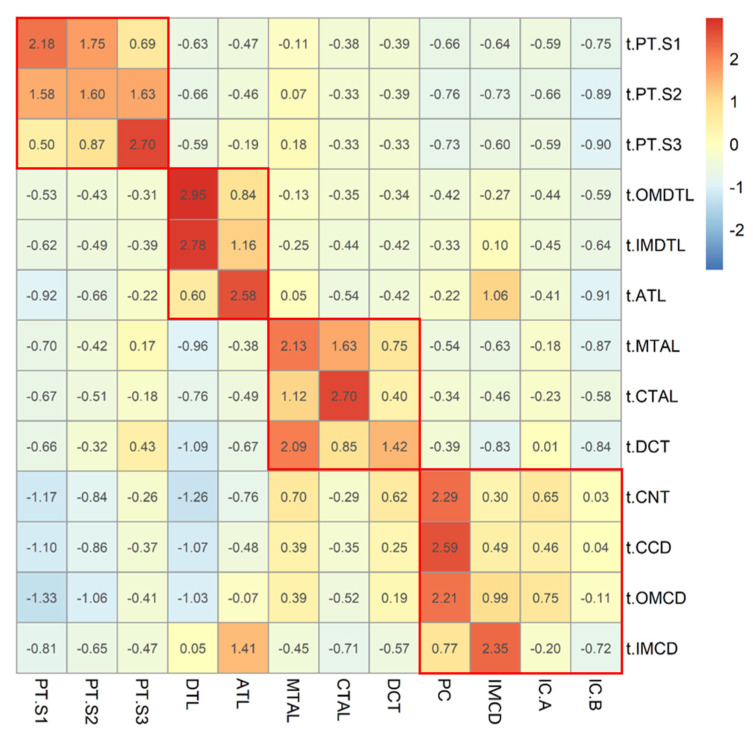
Correlation between rat scRNAseq clusters transcriptomes and bulk transcriptomes from rat microdissected nephron segments. A genomewide Pearson correlation was calculated and normalized. For each microdissected segment, the values on the table represent the number of standard deviations that the correlation with each cluster deviates from the average of all clusters. We used the “Median” gene expression of each segment as originally published by Knepper and collaborators. The use of the median gene expression value is robust against contamination with other segments as far as such contamination is present in less than 50% of the samples. Bulk transcriptomics of microdissected S1 segments primarily correlate with the PT.S1 scRNAseq cluster but also present a strong association with the PT.S2 scRNAseq cluster. Microdissected S2 segments present similar correlations with all three PT subsegment clusters. Microdissected S3 segments present the strongest correlation overall with the PT.S3 cluster. Microdissected segments of the loop of Henle present a strong correlation with the corresponding cell types characterized in the scRNAseq. Notably, microdissected DCT correlated with the DCT cluster expressing the sodium-chloride symporter (NCC, Slc12a3) but also with the MTAL cluster. The PC cluster dominates the correlations in CNT and collecting duct segments. Finally, the IMCD cell type was most highly correlated with the inner medullary collecting duct (IMCD) segment transcriptome.

**Figure A5:**
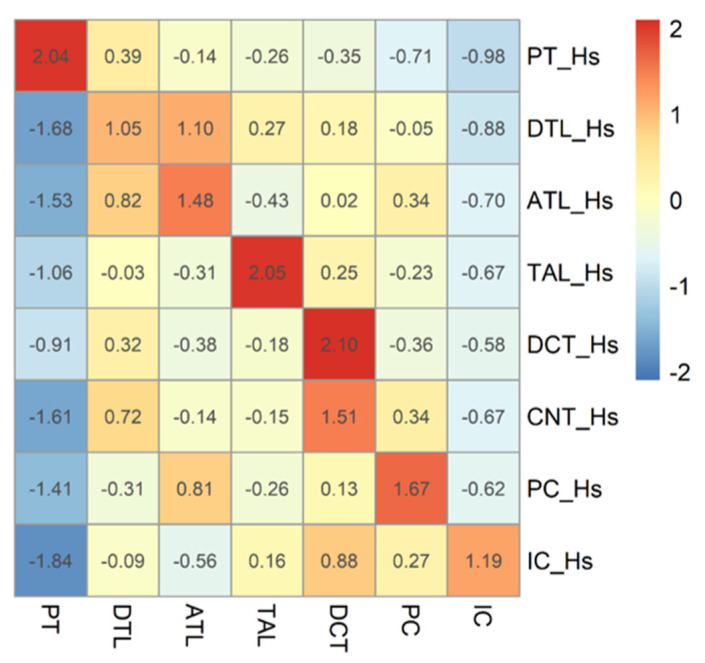
Correlation between rat scRNAseq cell cluster regions and transcriptomes from human kidneys. Normalized Pearson correlation between rat cell clusters and human single-nucleus (sn)RNAseq sub-regional transcriptomes. Of note, the rat scRNAseq dataset contains nearly 10% of the cell count of the KPMP snRNAseq and with less sequencing depth. As such the tubular epithelial clusters identified in the rat (horizontal axys), provided enough resolution for comparison with the subRegion (subclass.l1, vertical axys) but not the cell type (subclass.l2) identified in humans.

**Figure A6:**
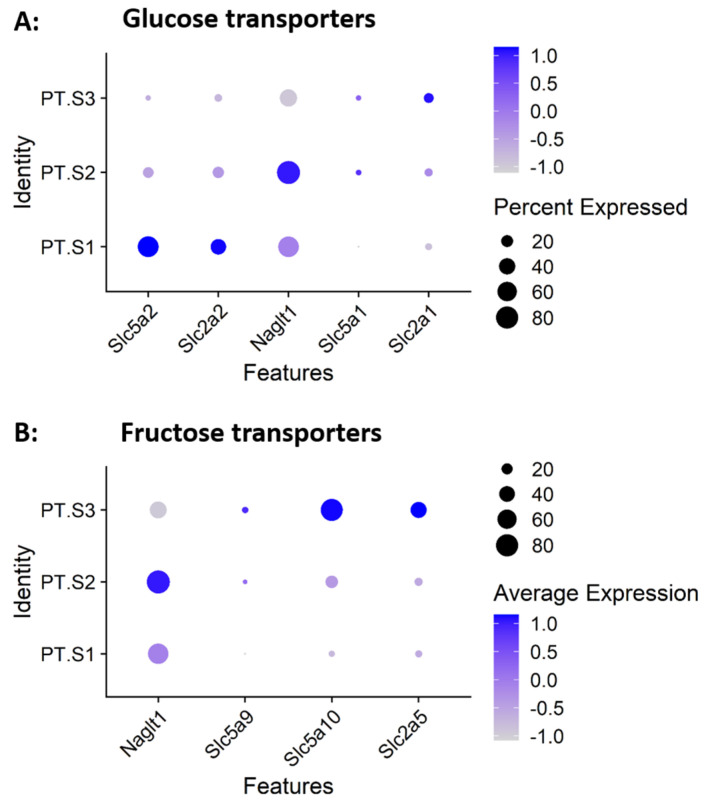
Normalized expression of sugar transporters in rat scRNAseq proximal tubule clusters. (**A**) Glucose transporters and (**B**) fructose transporters.

**Figure A7:**
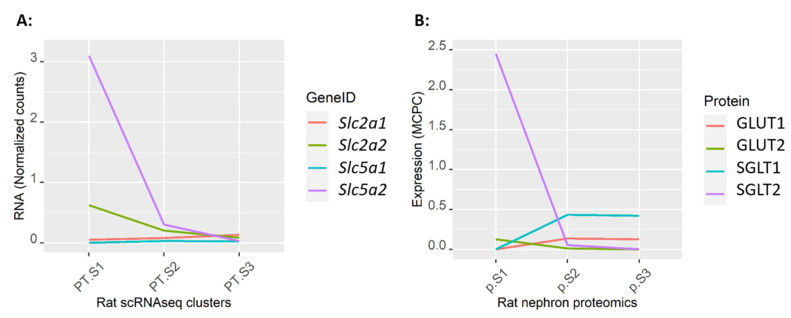
Normalized RNA transcripts of glucose transporters in rat scRNAseq proximal tubule clusters (**A**) compared to their corresponding gene products expressed in “million copies per cell” (MCPC) in microdissected rat proximal tubule segments (**B**). The order and colors of GeneIDs on the left panels match that of the corresponding gene products (Protein) on the right panels. Glucose and fructose transporter NAGLT1 are not shown in this figure, as it has already been shown in Figure 5 of the main manuscript.

**Figure A8:**
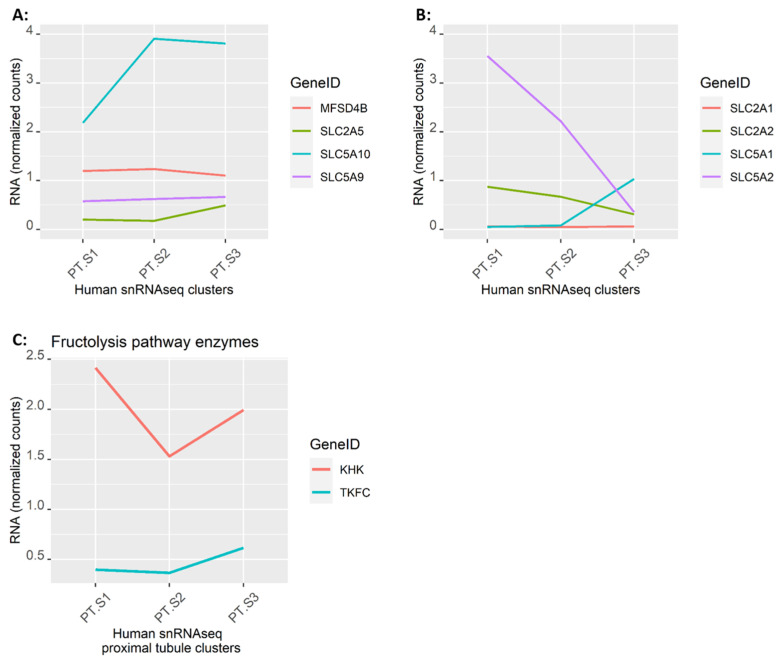
Normalized RNA transcript expression in snRNAseq proximal tubule clusters from human kidneys. (**A**) Fructose transporters, (**B**) glucose transporters, and (**C**) fructokinase (KHK) and triokinase (TKFC).

The genomewide Pearson correlation between pseudobulk transcriptomes from rat clusters and KPMP snRNAseq cell classes was used to identify and extract epithelial cells (Appendix A in Figure A1). Clusters were manually assigned to different kidney cell types using marker genes from the HuBMAP Kidney v1.2 cell markers [21] dataset, and other sources [22,23] (Appendix A in Figure A2). Clusters 17 and 27 presented low and diffuse expression of tubular epithelial cell markers and were therefore excluded from subsequent analysis. The expression of glomerular visceral epithelial cell (podocyte) markers was not distinguishable from background levels, while the expression of different PEC markers was scattered across multiple clusters (Appendix A in Figure A3). This indicates a low recovery of glomerular cells, unable to form separate clusters.

### 4.7. Digital Cytometry of Rat Microdissected Tubule Segments Transcriptomes

CibersortX [76] was used to estimate the fractional composition of distinct transcriptional phenotypes within the kidney tubule segments. Bulk transcriptomes from rat micro-dissected nephron segments were deconvoluted using the scRNAseq clusters transcriptomes generated here as a reference. In brief, raw sequencing files from both datasets were downloaded from the Sequence Read Archive (SRA) and reprocessed as described above. The number of detected genes was 34,322 for the bulk RNAseq data and 15,282 for the scRNAseq pseudobulk transcriptomes, with a shared gene space of 13,373 that was used for deconvolution. CibersortX was run with predetermined parameters, and without permutations for significance testing to focus solely on estimating cell type fractions.

### 4.8. Rat Microdissected Tubule Segments Proteomics

Quantitative proteomics data from rat PT subsegments [45] were downloaded from the National Heart, Lung, and Blood Institute (NHLBI-NIH) Epithelial Systems Biology Laboratory [77].

## 5. Conclusions

The results of this study imply that the S3 subsegment of the proximal tubule displays the highest capacity to reabsorb fructose in both humans and rats. Furthermore, data showing diverse cell populations within each segment of the nephron could aid in the interpretation of anatomical labels assigned to scRNAseq clusters.

## Data Availability

The raw data utilized for this study are openly available in multiple repositories. Accession numbers or URLs are provided for each data type in the Materials and Methods section. The code used to generate the Seurat object and the count matrix from micro-dissected nephron segments has been deposited in https://github.com/AgustinGonvi/Fructose-transporters-expression-in-proximal-tubules. Any new data generated to support the conclusions of this article will be made available by the authors upon request.

## Supplementary Materials

The following supporting information can be downloaded at: https://www.mdpi.com/article/10.3390/ijms25053071/s1 [78].
