# Supplementary material for: Profiling Cell Heterogeneity and Fructose Transporter Expression in the Rat Nephron by Integrating Single-Cell and Microdissected Tubule Segment Transcriptomes
Source: Int J Mol Sci. 2024 Mar 6;25(5):3071. doi: 10.3390/ijms25053071 (PMC10931557; doi:10.3390/ijms25053071)
Supplement: Supplementary file 1 [file ijms-25-03071-s001.zip › Supplemental Table 1.pdf]

Transporter		Substrate affinity (km; mmol/l)			Sugar:Na$^+$ coupling	References
(Protein)	(Gene)	Glucose	Fructose	Na$^+$		
SGLT5	Slc5a10	10.1	0.62	36	1:1	[1, 2, 3]
NaGLT1	Naglt1	3.7	~6.2	202	1:1	[4, 5]
GLUT5	Slc2a5	-	~9.3	-	-	[6]
SGLT4	Slc5a9	7.7	> 10; ~100 (?)	na.	na.	[3, 7]

References: (1-7)

1. **Ghezzi C, Gorraitz E, Hirayama BA, Loo DD, Grempler R, Mayoux E, and Wright EM**. Fingerprints of hSGLT5 sugar and cation selectivity. *Am J Physiol Cell Physiol* 306: C864-870, 2014.

2. **Grempler R, Augustin R, Froehner S, Hildebrandt T, Simon E, Mark M, and Eickelmann P**. Functional characterisation of human SGLT-5 as a novel kidney-specific sodium-dependent sugar transporter. *FEBS Lett* 586: 248-253, 2012.

3. **Diederich J, Mounkoro P, Tirado HA, Chevalier N, Van Schaftingen E, and Veiga-da-Cunha M**. SGLT5 is the renal transporter for 1,5-anhydroglucitol, a major player in two rare forms of neutropenia. *Cell Mol Life Sci* 80: 259, 2023.

4. **Horiba N, Masuda S, Takeuchi A, Takeuchi D, Okuda M, and Inui K**. Cloning and characterization of a novel Na+-dependent glucose transporter (NaGLT1) in rat kidney. *J Biol Chem* 278: 14669-14676, 2003.

5. **Horiba N, Masuda S, Ohnishi C, Takeuchi D, Okuda M, and Inui K**. Na(+)-dependent fructose transport via rNaGLT1 in rat kidney. *FEBS Lett* 546: 276-280, 2003.

6. **Nomura N, Verdon G, Kang HJ, Shimamura T, Nomura Y, Sonoda Y, Hussien SA, Qureshi AA, Coincon M, Sato Y, Abe H, Nakada-Nakura Y, Hino T, Arakawa T, Kusano-Arai O, Iwanari H, Murata T, Kobayashi T, Hamakubo T, Kasahara M, Iwata S, and Drew D**. Structure and mechanism of the mammalian fructose transporter GLUT5. *Nature* 526: 397-401, 2015.

7. **Tazawa S, Yamato T, Fujikura H, Hiratochi M, Itoh F, Tomae M, Takemura Y, Maruyama H, Sugiyama T, Wakamatsu A, Isogai T, and Isaji M**. SLC5A9/SGLT4, a new Na+-dependent glucose transporter, is an essential transporter for mannose, 1,5-anhydro-D-glucitol, and fructose. *Life Sci* 76: 1039-1050, 2005.
